# Supplementary material for: Evaluating the risk of data loss due to particle radiation damage in a DNA data storage system
Source: Nat Commun. 2024 Sep 14;15:8067. doi: 10.1038/s41467-024-51768-x (PMC11401870; doi:10.1038/s41467-024-51768-x)
Supplement: Supplementary file 1 — Supplementary Information [file 41467_2024_51768_MOESM1_ESM.pdf]

Evaluating the Risk of Data Loss Due to Particle Radiation Damage in a DNA Data Storage System

Christopher N. Takahashi[1], David P. Ward[1], Carlo Cazzaniga[2], Christopher Frost[2], Paolo Rech[3], Kumkum Ganguly[4], Sean Blanchard[4], Steve Wender[4], Bichlien H. Nguyen[1,5*] and Jake A. Smith[1,5*]

[1]Paul G. Allen School of Computer Science and Engineering, University of Washington, Seattle, WA, USA.
[2]Science and Technology Facilities Council, Swindon, United Kingdom.
[3]University of Trento, Trento, Italy.
[4]Los Alamos National Laboratory, Los Alamos, NM, USA.
[5*]Microsoft Research, Redmond, WA, USA.

*Corresponding author(s). E-mail(s): bnguy@microsoft.com; jakesmith@microsoft.com;

Contents

1 Regression Reports **2**

2 Supplementary Figures **4**

3 Monte Carlo Simulation **5**

1 Regression Reports

σ^n_{nuc} Fitted With Sample Thickness Term

$$\log(\sigma^n) = \begin{cases} 0 & \log_{10}(E) < 6 \\ \beta_0 + \beta_1 \log_{10}(E) + \beta_2 \log_{10}(E)^2 + \beta_3 \log_{10}(E)^3 + \beta_4 \log_{10}(L_Z) & 6 \leq \log_{10}(E) \leq 10 \\ 0 & \log_{10}(E) > 10 \end{cases}$$

**Dep. Variable:**	y	**No. Observations:**	160
**Model:**	Poisson	**Df Residuals:**	155
**Method:**	MLE	**Df Model:**	4
**Date:**	Fri, 15 Dec 2023	**Pseudo R-squ.:**	0.5226
**Time:**	12:40:57	**Log-Likelihood:**	-244.96
**converged:**	True	**LL-Null:**	-513.13
**Covariance Type:**	nonrobust	**LLR p-value:**	9.281e-115

	**coef**	**std err**	**t**	**P>** t	**[0.025**	**0.975]**
β_1	72.9389	10.428	6.995	0.000	52.340	93.538
β_2	-8.5132	1.332	-6.389	0.000	-11.145	-5.881
β_3	0.3223	0.056	5.715	0.000	0.211	0.434
β_4	-0.0192	0.035	-0.548	0.585	-0.089	0.050
β_0	-201.7467	27.038	-7.462	0.000	-255.158	-148.336

σ^n_{H2O} Fitted With Sample Thickness Term

$$\log(\sigma^n) = \begin{cases} 0 & \log_{10}(E) < 6 \\ \beta_0 + \beta_1 \log_{10}(E) + \beta_2 \log_{10}(E)^2 + \beta_3 \log_{10}(E)^3 + \beta_4 \log_{10}(L_Z) & 6 \leq \log_{10}(E) \leq 10 \\ 0 & \log_{10}(E) > 10 \end{cases}$$

**Dep. Variable:**	y	**No. Observations:**	160
**Model:**	Poisson	**Df Residuals:**	155
**Method:**	MLE	**Df Model:**	4
**Date:**	Fri, 15 Dec 2023	**Pseudo R-squ.:**	0.2322
**Time:**	13:44:04	**Log-Likelihood:**	-56.592
**converged:**	True	**LL-Null:**	-73.704
**Covariance Type:**	nonrobust	**LLR p-value:**	6.704e-07

	**coef**	**std err**	**t**	**P>** t	**[0.025**	**0.975]**
β_1	144.5223	59.905	2.413	0.017	26.188	262.857
β_2	-17.1974	7.464	-2.304	0.023	-31.942	-2.453
β_3	0.6710	0.308	2.178	0.031	0.062	1.280
β_4	0.0519	0.162	0.320	0.750	-0.269	0.373
β_0	-400.3496	159.323	-2.513	0.013	-715.074	-85.625

σ^n_{nuc} Fitted With Fraction GC Content Term

$$\log(\sigma^n) = \begin{cases} 0 & \log_{10}(E) < 6 \\ \beta_0 + \beta_1 \log_{10}(E) + \beta_2 \log_{10}(E)^2 + \beta_3 \log_{10}(E)^3 + \beta_4 F_{GC} & 6 \leq \log_{10}(E) \leq 10 \\ 0 & \log_{10}(E) > 10 \end{cases}$$

**Dep. Variable:**	y	**No. Observations:**	160
**Model:**	Poisson	**Df Residuals:**	155
**Method:**	MLE	**Df Model:**	4
**Date:**	Fri, 15 Dec 2023	**Pseudo R-squ.:**	0.5234
**Time:**	13:48:33	**Log-Likelihood:**	-244.55
**converged:**	True	**LL-Null:**	-513.13
**Covariance Type:**	nonrobust	**LLR p-value:**	6.124e-115

	**coef**	**std err**	**t**	**P>** t	**[0.025**	**0.975]**
**x1**	31.8624	4.542	7.015	0.000	22.890	40.835
**x2**	-1.6153	0.252	-6.409	0.000	-2.113	-1.117
**x3**	0.0266	0.005	5.735	0.000	0.017	0.036
**x4**	1.9635	1.821	1.078	0.283	-1.633	5.560
**const**	-203.6525	27.153	-7.500	0.000	-257.290	-150.015

σ^n_{H2O} Fitted With Fraction GC Content Term

$$\log(\sigma^n) = \begin{cases} 0 & \log_{10}(E) < 6 \\ \beta_0 + \beta_1 \log_{10}(E) + \beta_2 \log_{10}(E)^2 + \beta_3 \log_{10}(E)^3 + \beta_4 F_{GC} & 6 \leq \log_{10}(E) \leq 10 \\ 0 & \log_{10}(E) > 10 \end{cases}$$

**Dep. Variable:**	y	**No. Observations:**	160
**Model:**	Poisson	**Df Residuals:**	155
**Method:**	MLE	**Df Model:**	4
**Date:**	Fri, 15 Dec 2023	**Pseudo R-squ.:**	0.2341
**Time:**	13:51:05	**Log-Likelihood:**	-56.453
**converged:**	True	**LL-Null:**	-73.704
**Covariance Type:**	nonrobust	**LLR p-value:**	5.880e-07

	**coef**	**std err**	**t**	**P>** t	**[0.025**	**0.975]**
**x1**	61.3290	25.954	2.363	0.019	10.061	112.597
**x2**	-3.1682	1.405	-2.255	0.026	-5.943	-0.393
**x3**	0.0537	0.025	2.130	0.035	0.004	0.103
**x4**	-5.7263	9.559	-0.599	0.550	-24.609	13.157
**const**	-389.2173	159.205	-2.445	0.016	-703.708	-74.726

2 Supplementary Figures

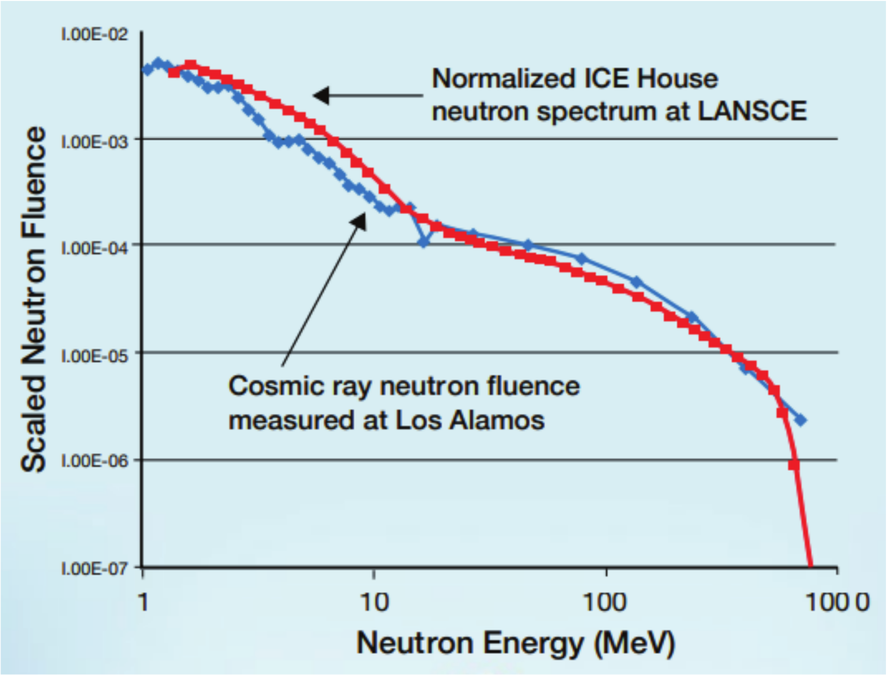

Fig. 1 Plot of the neutron energy spectrum measured at LANSCE ICE as compared to the background cosmic ray neutron fluence measured at LANSCE. Retrieved from the LANSCE ICE House testing manual.

3 Monte Carlo Simulation

The supplementary software file `run_simulation.py` was used for approximation of σ^n_{nuc} and σ^n_{H2O} by Monte Carlo simulation. To replicate, please take the following steps.

- Prepare a `Python` environment that includes the packages `numpy`, `pandas`, and `scipy`.
- Divide the pre-formatted data files found in tabs of the provided Excel sheet into a series of `.csv` files.
- Set an environmental variable `CS_DATA_DIR` containing the path to the separated data files.
- For a given combination of neutron energy and target DNA depth, execute `run_simulation.py`.

```
python run_simulation.py <neutron_energy> <dna_depth> <output_path>
```
