# Supplementary material for: Evaluating the risk of data loss due to particle radiation damage in a DNA data storage system
Source: Nat Commun. 2024 Sep 14;15:8067. doi: 10.1038/s41467-024-51768-x (PMC11401870; doi:10.1038/s41467-024-51768-x)
Supplement: Supplementary file 3 — Description of Additional Supplementary Files [file 41467_2024_51768_MOESM3_ESM.pdf]

Title: Supplementary Software
Description: The script used to run Monte Carlo simulations.

Title: Supplementary Data 1
Description: Parameters used to run Monte Carlo simulations.
